# Locust Hemolymph Conveys Erythropoietin-Like Cytoprotection *via* Activation of the Cytokine Receptor CRLF3

**DOI:** 10.3389/fphys.2021.648245

**Published:** 2021-04-09

**Authors:** Debbra Y. Knorr, Denise Hartung, Kristin Schneider, Luzia Hintz, Hanna S. Pies, Ralf Heinrich

**Affiliations:** Department of Cellular Neurobiology, Johann-Friedrich-Blumenbach Institute for Zoology and Anthropology, Georg-August-University Göttingen, Göttingen, Germany

**Keywords:** cytokine receptor-like factor 3, hemolymph, cytokine, cytoprotection, neuroprotection, hemocytes, neurons, insect

## Abstract

The cytokine receptor-like factor 3 (CRLF3) is an evolutionary conserved class 1 cytokine receptor present in all major eumetazoan groups. Endogenous CRLF3 ligands have not been identified and the physiological responses mediated by mammalian CRLF3 are poorly characterized. Insect CRLF3 is activated by erythropoietin (Epo) and several related molecules that protect mammalian neurons from stress-induced apoptosis. However, insects neither express Epo nor “classical” Epo receptor. Cell-protective effects of insect hemolymph have been described for several species. In this study, we explored the possibility that the endogenous CRLF3 ligand is contained in locust hemolymph. PCR analyses confirmed expression of *crfl3*-transcripts in neurons and hemocytes of *Locusta migratoria* and *Tribolium castaneum*. Survival of locust hemocytes in primary cultures was significantly increased by supplementation of culture medium with locust hemolymph serum. Locust primary neuron cultures were also protected by locust hemolymph, though preceding exposure to fetal bovine serum changed the hemolymph dose-dependency of neuroprotection. Direct comparison of 10% hemolymph serum with recombinant human Epo in its optimal neuroprotective concentration revealed equivalent anti-apoptotic effects on hypoxia-exposed locust neurons. The same concentration of locust hemolymph serum also protected hypoxia-exposed *T. castaneum* neurons. This indicates that the neuroprotective factor in locust hemolymph is sufficiently conserved in insects to allow activation of neuroprotective receptors in different species. Locust hemolymph-induced neuroprotection in both *L. migratoria* and *T. castaneum* was abolished after RNAi-mediated suppression of *crlf3*-expression. In summary, we report the presence of a conserved endogenous cytokine in locust hemolymph that activates CRLF3 and connected anti-apoptotic processes in hemocytes and neurons. Identification and characterization of the CRLF3 ligand will promote knowledge about cytokine evolution and may unravel cell-protective agents with potential clinical application.

## Introduction

Insect hemolymph contains multiple cell types, suspended in a protein-rich liquid plasma, that circulates through the body cavity of the organism (reviewed by Douglas and Siva-Jothy, 2013; Hillyer and Pass, 2020). Propelled through an open circulatory system by a contractile dorsal vessel and accessory pulsatile organs, hemolymph directly contacts most insect tissues. However, metabolic exchange with the central nervous system is restricted and regulated by the “hemolymph-brain-barrier,” consisting of surface glia interconnected by septate junctions (reviewed by Weiler et al., 2017). Hemolymph and hemocyte functions are largely similar to those of vertebrate blood, including transport of metabolites and hormones, maintaining homeostasis (pH, osmolarity, water balance, and ion composition), sealing of wounds, and serving immune functions among others. In contrast to the mammalian acquired immunity involving specific antibodies and memory cells, insects rely on innate immune functions to neutralize various kinds of pathogens (Lavine and Strand, 2002; Siva-Jothy et al., 2005; Strand, 2008). Immune responses in mammals and insects are balanced by cytokines that either promote or dampen defensive cellular reactions. Hemolymph proteins involved in immune functions and adaptation to environmental challenges are synthesized and released into the circulation by the fat body, midgut, endocrine glands such as *corpora allata* or the *corpora cardiaca*, neurosecretory cells, hemocytes, and other organs (Arrese and Soulages, 2010; Oda et al., 2010; Roma et al., 2010; Hoshizaki, 2013; Kodrík et al., 2015).

Several hemolymph proteins directly interfere with invading pathogens (anti-microbial peptides; reviewed by Yi et al., 2014; Wu et al., 2018) or activate hemocytes to neutralize pathogens by various mechanisms (plasmatocyte spreading peptides, growth blocking peptides, and paralytic peptides; Strand, 2008; Duressa et al., 2015). Other hemolymph proteins initiate physiological adaptations of insect tissues, supporting cell survival and functionality in challenging conditions. Previous publications have reported protective and anti-apoptotic effects of insect hemolymph on various cell types. Studies with *Bombyx mori* hemolymph demonstrated beneficial effects on lepidopteran cells and various rodent and human cell lines (Rhee and Park, 2000; Rhee et al., 2002; Park et al., 2003; Kim et al., 2004; Yu et al., 2013). A group of approximately 30 proteins (named 30K protein family due to their molecular sizes around 30 kDa) was identified to promote cell survival, with individual proteins contributing some portion of the protective effects (Kim et al., 2001, 2003, 2004; Rhee et al., 2002; Park et al., 2003; Zhong et al., 2005; Yu et al., 2013; Pakkianathan et al., 2015). Protective effects of these 30K proteins seem to be mediated (at least to a large extent) by adhesion to cellular membranes rather than by activation of a particular receptor. In addition to lepidopteran species, beneficial effects of hemolymph on the survival and neurite regeneration of insect neurons have been reported in locusts and cockroaches (Howes et al., 1991; Kirchhof and Bicker, 1992).

Cytokines are involved in responses to exogenous and endogenous insults, repair and restoration of tissue homeostasis in both invertebrates and vertebrates (Beschin et al., 2001; Liongue and Ward, 2007). Various insect cytokines and cytokine-like factors have been identified (e.g., growth blocking peptide, spätzle, unpaired, vago, insect chemotactic peptide, stress responsive peptide, and diedel) to activate specific cytokine receptors (e.g., domeless, toll, and growth blocking peptide receptor) or interact with cell surface carbohydrate patterns (Zanetta et al., 1996; Welchman et al., 2009; Ghezzi and Conklin, 2013; Kingsolver et al., 2013; Tsuzuki et al., 2014; Lamiable et al., 2016). Though cytokines typically share little sequence similarities between animal groups (Liongue and Ward, 2007), some invertebrate cytokines can activate mammalian cytokine receptors, e.g., sponge-derived cytokines activate thrombopoietin receptor and/or erythropoietin (Epo) receptor (Watari et al., 2019). Particular cytokines often activate different receptors, receptors may be activated by multiple cytokine ligands and cytokine receptor diversity is increased by formation of homo- and heteromeric receceptor complexes with different stoichiometry of subunits (Liongue et al., 2016). The flexible or loose match between cytokines and cytokine receptors within and across species appears to be a common characteristic of cytokine signaling that may result from a common evolutionary origin (Huising et al., 2006).

We have recently reported the expression of the cytokine receptor, cytokine receptor-like factor 3 (CRLF3), in hemocytes of *Locusta migratoria* (Hahn et al., 2019). CRLF3 is an orphan class I cytokine receptor that shares similarities with vertebrate receptors for prolactin, growth hormone, thrombopoietin, and Epo (Boulay et al., 2003; Liongue and Ward, 2007). While most class 1 cytokine receptors are exclusively present in vertebrates, CRLF3 is highly conserved throughout eumetazoan species ranging from cnidarians to humans (Hahn et al., 2019). Despite the absence of Epo or recognized Epo receptors in insects, recombinant human Epo (rhEpo) protects cultured insect neurons from apoptotic cell death (Miljus et al., 2014; Hahn et al., 2017; Heinrich et al., 2017) and promotes regeneration of neurites *in vitro* and *in vivo* (Ostrowski et al., 2011). CRLF3 was identified as the neuroprotective receptor activated by rhEpo in the orthopteran *L. migratoria* and the coleopteran *Tribolium castaneum* (Hahn et al., 2017, 2019). Similar to Epo-mediated neuroprotection in vertebrates (Brines and Cerami, 2005; Zhang et al., 2014; Ostrowski and Heinrich, 2018), anti-apoptotic effects in insect neurons are mediated by janus kinase/signal transducer and activator of transcription (JAK/STAT) transduction (Miljus et al., 2014). Activation of insect CRLF3 by human Epo and several other molecules that mimic its neuroprotective functions in mammals indicates structural similarities between the ligand-binding domains of mammalian tissue-protective Epo receptors and insect CRLF3. Nonetheless, CRLF3 is still considered as an orphan receptor since its endogenous ligand has not been identified in any organism expressing the receptor. Expression of CRLF3 in insect brains, muscle, and hemocytes (Hahn et al., 2019) parallels the multi-tissue-expression of its mammalian orthologues (Yang et al., 2009) and suggests both local tissue-specific and systemic functions. The endogenous ligand must be present in both the nervous system and the hemolymph, suggesting its production on both sides of the hemolymph-brain-barrier. The ligand, like Epo in mammals, might activate CRLF3 in various tissues to mediate general cell protection.

In the present study, we investigated the cytoprotective potential of locust hemolymph by using the previously described protective effects of rhEpo as comparison. We show expression of *crfl3* in brain and hemocytes of *L. migratoria* and *T. castaneum*. *In vitro* experiments on primary cell cultures reveal dose-dependent anti-apoptotic effects of cell-free *L. migratoria* hemolymph on locust hemocytes and neurons. Locust hemolymph also protected *T. castaneum* neurons. Neuroprotection in both locust and beetle neurons was mediated by CRLF3 and was similar to previously reported rhEpo protection. These results indicate the presence of a conserved CRLF3 ligand in insect hemolymph that initiates protective mechanisms in different cell types.

## Materials and Methods

Studies were performed with the migratory locust *L. migratoria* and the red flour beetle *Tribolium castaneum*. Locusts were purchased from a commercial breeder (HW-Terra; Herzogenaurach, Germany) and maintained at 24°C; 55% humidity with a 12/12 h day/night cycle. *T. castaneum* were maintained on full grain flour and yeast in plastic boxes at 27°C and 40% humidity.

### Hemolymph Extraction From Locusts

Adult locusts were cooled at 4°C for 10 min. Cold-anesthetized animals were fixed dorsal side down on clay without injuring the animal. Seven-hundred microliter ice cold anticoagulation solution (ACS; 98 mM NaOH, 186 mM NaCl, 17 mM Na_2_EDTA, 41 mM citric acid, pH 4.5) was slowly injected into the lower abdomen. After incubating for 1 min, a small incision was made at the injection site and the hemolymph (~1 ml) was extracted with a Pasteur pipette. Collected hemolymph (HL) and ACS mix was transferred to an Eppendorf tube containing 500 μl ice cold ACS. The mixture was centrifuged at 500 x *g* for 5 min. The cell-free serum was transferred to a fresh Eppendorf tube. HL of different animals (up to 100) was pooled and sterile filtered (0.20 μm syringe filter; Merck, Darmstadt, Germany) twice to ensure sterility and to avoid conglomerates in the sample.

In order to separate proteins of HL samples from ACS, the HL/ACS mixture was purified by molecular weight cut off (MWCO) filters (Cut off 5,000 Da; Corning, New York, United States). HL/ACS was transferred into sterile (sterilized through 24 h UV-light exposure) MWCO filters and centrifuged at 4000 x *g* for up to 3 h until samples were highly concentrated. To ensure full ACS elimination from samples, concentrated HL was diluted 1:5 in phosphate-buffered saline (PBS) and respun in MWCO filters. This procedure was repeated four times. Samples of HL/ACS extract, MWCO filter flow through and purified HL containing hemolymph proteins were analyzed by 10% sodium dodecyl sulfate–polyacrylamide gel electrophoresis (SDS-PAGE) electrophoresis. Filter flow through contained no significant amounts of protein (data not shown) indicating little loss of proteins during the purification and buffer exchange procedure. Purified HL was aliquoted á 100 μl and stored at −20°C until further usage as experimental supplement to cell culture medium.

### Heat Denaturation of HL

To evaluate if cell protective effects of HL were due to a protein or a peptide, HL was subjected to heat denaturation. Eppendorf cups containing 1 ml HL were immersed in a temperature-controlled water bath at either 63 or 103°C (to compensate for isolation by the Eppendorf cup) for 10 min. Denatured HL (dHL) was centrifuged for 10 min at 1000 x *g* in order to spin down precipitated proteins. Supernatant was transferred to a fresh tube and cooled on ice. dHL was stored at −20°C until further usage.

### Hemocyte Culture

Adult locusts were cold-anesthetized for 10 min at 4°C. Cell culture plates (Ø 3 cm; Corning, New York, United States) were equipped with 1 cm coverslips (Hartenstein, Würzburg, Germany). Coverslips were coated with Concanavalin A (Sigma-Aldrich, Munich, Germany) for 1 h and subsequently washed three times with PBS. Each experiment contained hemocytes of only one animal that were allocated to differently treated cultures.

Hemolymph was collected from cooled locusts and hemocytes were separated from serum by centrifugation as described above. Serum was discarded and the cell pellet was resuspended in 1 ml sterile ACS. Cell suspension was centrifuged for 5 min. Supernatant was discarded and hemocytes were resuspended in 1 ml basal Grace insect medium (Gibco, Life Technologies, Darmstadt, Germany) +5% penicillin/streptomycin (P/S; 10,000 units/ml penicillin and 10 mg/ml streptomycin, Sigma-Aldrich, Munich, Germany) +5% Amphotericin B (AmphoB; Gibc, 250 μg/ml, ThermoFisher Scientific, Osterode am Harz, Germany). Cells were centrifuged and supernatant was discarded. The cell pellet was resuspended in 1 ml Grace + P/S + AmphoB and 10 μl of cell suspension was used for cell counting with a Neubauer Improved counting chamber (0.1 mm depth; Marienfeld Superior, Lauda-Königshofen, Germany). Cell suspension was centrifuged one last time and resuspended in fresh medium. 25,000 cells per coverslip were seeded. Cells were let to settle and attach to the coverslip for 2 h before dishes were filled with 500 μl cell culture medium. Cultures were maintained at 27°C in normal atmosphere. First medium change was performed the next day, subsequently medium was exchanged every 2nd day.

### Treatment of Hemocyte Cultures

Hemocyte cultures were established as described above. Right after establishment, hemocyte cultures were maintained in either basal Grace insect medium, 100% HL, 50% HL mixed with Grace medium or in Grace medium supplemented with 33.3 ng/ml recombinant human Epo (rhEpo/Epo). Cells were maintained for 7 days (respective culture medium was renewed on *in vitro* day 1, 3, and 5), fixed in 4% paraformaldehyde and prepared for cell survival analysis (see below).

### Locust and Beetle Neuron Culture

Primary neuron cultures were established from 5th instar locust nymphs (previously described by Miljus et al., 2014; Hahn et al., 2019; Knorr et al., 2020) or late beetle pupae (previously described by Hahn et al., 2017). In brief, two locust brains or 20 beetle brains per culture were dissected. Brains were washed three times in Leibowitz 15 medium (L15; Gibco, Life Technologies, Darmstadt, Germany) and supplemented with 1% P/S and 1% AmphoB. L15 with antibiotics lacking any additional additives will be referred to as “basal L15 medium” throughout the manuscript. Insect brains were then transferred to an enzyme mix containing Collagenase/Dispase (2 mg/ml; Sigma-Aldrich, Munich, Germany) for 30 min (locust) or 45 min (beetle) at 27°C. Digestion was stopped by washing the brains three times in Hanks balanced salt solution (Gibco, Life Technologies, Darmstadt, Germany). Subsequently, brains were mechanically dissociated by repeated pipetting until no chunks of tissue remained. Dissociated neurons were centrifuged down, washed once with medium, and centrifuged again. Cells were resuspended in 100 μl/coverslip cell culture medium and plated on Concanavalin A coated 1 cm coverslips. Cells were let to rest for 2 h and dishes were subsequently filled up with medium supplemented with 5% fetal bovine serum gold (FBSG/FBS; PAA Laboratories GmbH, Pasching, Austria), a natural serum with individual ingredients first separated and then recomposed in a defined composition. All experiments were performed with the same Lot of FBS. Cells were maintained at 27°C in normal atmosphere and medium was changed every other day.

### Dose-Dependent HL Effect on Neuronal Survival

To determine potential effects of HL on locust *in vitro* neuronal survival, HL was mixed in different proportions with culture medium. Four primary neuron cultures per experiment were established as described above and kept at 27°C throughout the experiment. Neuron cultures were physiologically challenged to different degrees in three variations of the experimental protocol.

#### Cell Survival in Unchallenged Conditions

All neuron cultures were initially cultured in basal L15 medium with 5% FBSG supplementation for 2 days, to support their transition to *in vitro* conditions. After this initial period, cultures were exposed for 3 days to basal L15 medium without FBSG (control) or culture medium with either 10, 25, or 50% HL supplementation. After 5 days *in vitro*, neurons were fixed and prepared for survival analysis.

#### Cell Survival Without Initial FBSG Treatment

Neuron cultures were maintained in basal L15 medium for 2 days. Subsequently cells were treated with basal L15 medium (control) or either 10, 25, or 50% HL in L15 for another 3 days before fixation and analysis.

#### Cell Survival in Hypoxia

Cultures were established as described above and maintained in medium supplemented with 5% FBSG for 4 days. On *in vitro* day 5, cultures were treated with basal L15 medium (hypoxia control) or either 50, 25, 10% HL or 33.3 ng/ml Epo diluted in L15 medium before being exposed to hypoxia 12 h later. One additional culture in basal L15 medium remained in standard atmospheric conditions (normoxia control). Neurons were maintained in hypoxic conditions (O_2_ < 0.3%; Hypoxia Incubator Chamber, STEMCELL™, Cologne, Germany) for 36 h. Subsequently cells were fixed and prepared for survival analysis.

Experiments with neuron cultures from *T. castaneum* were performed in a similar way; however, six differently treated cultures were compared in each experiment. On day 5 *in vitro* (12 h before being exposed to hypoxia for 36 h), three cultures were treated with either 0.25, 1, or 10% locust HL diluted in basal L15 medium. A fourth culture was treated with 3.33 ng/ml Epo. Normoxic and hypoxic control cultures in basal L15 medium were run for comparison with treated cultures as described above.

### Involvement of CRLF3 in HL-Mediated Neuroprotection

To analyze if neuroprotective effects of HL were mediated by CRLF3, soaking RNAi was performed on primary neuron cultures to knockdown either locust or beetle *crlf3* expression. Soaking RNAi was established previously for both species used in this study (Hahn et al., 2017, 2019). In the original publications, we validated the specificity of the knockdown by targeting two non-overlapping fragments of locust *Lm-crlf3* and beetle *Tc-crlf3* with different dsRNA molecules. In this study, *Lm-crlf3* Fragment 1 and *Tc-crlf3* Fragment 2 from the previous studies were used. Four cultures of either locust or beetle neurons were established as described above. One culture was treated with dsRNA (10 ng/μl) targeting *crlf3* immediately after culture establishment. FBSG was removed from cell culture media on day 4. On day 5, two cultures, one untreated and one dsRNA-treated, were exposed to 10% HL diluted in basal L15 medium. Twelve hours later, the treated cultures and one untreated culture (hypoxia control) were exposed to hypoxia (O_2_ < 0.3%) for 36 h. One additional neuron culture was kept in normoxic conditions (normoxia control) for the same time. Cells were subsequently fixed and analyzed for cell survival.

### Effects of Heat-Denatured HL on Neuron Survival

To evaluate if cell-protective effects were retained after heat denaturation of HL, neuron cultures were established, maintained, and exposed to hypoxia as described above. Five cultures were established and maintained in basal L15 medium supplemented with 5% FBSG for 4 days *in vitro*. Four cultures were treated with basal L15 medium (hypoxia control) or basal medium supplemented with either 10% HL, 10% dHL after exposure to 60°C or 10% dHL after exposure to 100°C, respectively, on day 5, 12 h before onset of hypoxia (O_2_ < 0.3%) for 36 h. One additional neuron culture was kept in normoxic conditions (normoxia control) for the same time. Cells were subsequently fixed and prepared for cell survival analysis.

### Cell Survival Assessment

Both hemocyte and neuron survival was analyzed as described previously (Miljus et al., 2014; Hahn et al., 2017, 2019). After fixation, coverslips with attached cells were washed (5 min per step) three times in PBS followed by two wash steps in PBS/0,1% Triton-X-100 (PBST). Cells were stained with Dapi (1:1000 in PBST; Sigma-Aldrich; Munich, Germany) for 30 min in the dark. Subsequently, coverslips were washed five times in PBS before mounting on microscopy slides in DABCO (Roth, Karlsruhe, Germany).

Coverslips were imaged with an epifluorescence microscope (Zeiss Axioskop; Oberkochen, Germany; 40x objective was used for locust neurons or hemocytes, 63x oil objective was used for tribolium neurons) equipped with a Spot CCD camera (Invisitron, Puchheim, Germany). Non-overlapping series of photographs passing the center of the coverslip to the left and the right were taken from all cultures (~80 pictures per locust culture and ~120 pictures per tribolium culture). Cells were manually scored as intact or dead/dying on the basis of Dapi-fluorescence pattern reflecting nuclear chromatin structure. The scorer was blinded with respect to the culture treatment during counting. Cell counting was supported by ImageJ Cell counter plug-in (Fiji ImageJ by NIH) as described elsewhere (Miljus et al., 2014; Hahn et al., 2019; Knorr et al., 2020).

### Statistical Analysis

Ratios of the numbers of intact and dead/dying cells of individual cultures were normalized to the respective untreated control cultures of the same experiment, providing the relative portion of surviving cells within the experiment. Data were analyzed using R studio Version 1.2.1335 (RStudio Team, 2015; R Core Team, 2019) employing pairwise permutation test included in packages “coin” and “rcompanion” (Hothorn et al., 2006, 2008; Mangiafico, 2019). Data are presented in box plots displaying the upper and lower quartiles and the medians. Whiskers represent 1.5x interquartile range. Single data points are shown by circles. Benjamini-Hochberg correction was applied to avoid false positives resulting from multiple comparisons.

### SDS-PAGE

To visualize the protein composition of HL, SDS-PAGE analysis was performed. Protein concentrations were measured by Bradford assay (Bradford solution; PanReac AppliChem, Darmstadt, Germany). For all samples, 50 μg protein was denatured at 75°C for 10 min in 2X Lämmli buffer (Sigma-Aldrich, Munich, Germany). Ten percent acrylamide (Sigma-Aldrich, Munich, Germany) gels were cast and samples were run in the Bio-Rad™ Mini Protean System (Bio-Rad™, Feldkirchen, Germany) for 30 min at 70 V followed by 60 min at 120 V. PageRuler Plus Prestained Protein ladder (Thermo Fisher Scientific, Osterode am Harz) was used as size reference. Gels were stained in InstantBlue™ Coomassie Protein Stain (Abcam, Cambridge, United Kingdom) over night and imaged using the iBright CL1500 Imaging System (Thermo Fisher Scientific, Osterode am Harz, Germany). SDS-PAGEs were run as controls for HL purification and for heat denatured HL to validate protein denaturation.

### RT-PCR

Locust and *T. castaneum* brains and locust hemocytes were extracted and collected as described above. *T. castaneum* hemocytes were collected from pupae by puncturing their lower abdomen. Forty punctured pupae were transferred to 0.5 ml Eppendorf cups with small holes in their bottom. This cup was then placed into a larger 1.5 ml Eppendorf cup containing 10 μl ACS. This composition was centrifuged for 10 min at 12,000 x *g* in order to collect the hemolymph in the bigger cup. Hemolymph diluted in ACS was then centrifuged at 5000 x *g* for 10 min to spin down hemocytes. Serum was discarded and hemocytes were subjected to RNA isolation. RNA from all cell types studied was isolated by a modified Trizole (Sigma-Aldrich, Munich, Germany) protocol (described by Knorr et al., 2020). In brief, tissue was disrupted in Trizole reagent, aided by the Tissue Lyser LT (Qiagen, Hilden, Germany) and a 3 mm stainless steel bead. Two-hundred microliter chloroform (Labsolute, Th. Geyer, Renningen, Germany) was added and samples were incubated for 15 min on ice. Samples were subsequently centrifuged for 15 min at 12,000 x *g* at 4°C. The resulting translucent phase was transferred to a fresh Eppendorf cup and mixed with ice cold 75% EtOH. Samples were then incubated at −20°C for at least 1 h before centrifuging for 10 min at 10,000 x *g*. RNA pellets were washed three times in 75% ice cold EtOH before airdrying. RNA concentrations were measured using NanoDrop 1,000 (Thermo Scientific, Schwerte, Germany). One microgram RNA was transcribed into cDNA using the LunaScript™ RT SuperMix Kit (New England BioLabs, Ipswich, MA, United States) according to the manufacturer’s instructions.

Reverse transcription polymerase chain reactions (RT-PCRs) were run for amplification of both tribolium and locust *crlf3*. Locust *18S rRNA* and tribolium α-*tubulin* were amplified as housekeeping genes (primer sequences listed in Table 1; *Lm*-*crlf3* was previously published in Hahn et al., 2019, *Lm*-*18s* was published in Knorr et al., 2020, and *Tc*-*crlf3* was published in Hahn et al., 2017). (−)RT controls were always run together with genes of interest. RT-PCR (program in Table 2) was run with GoTaq Green Master Mix (Promega, Germany) in a final reaction volume of 25 μl.

**Table 1:** Oligonucleotides used in this study.

*Gene*	*Sequence 5' – 3' FWD*	*Sequence 5' – 3' REV*	*Accession no*
*Lm*-*crlf3*	GGAACCAG*TC*AC*TC*TGCGAG	CGAATATTACCCCAGGCTGGAG	MN245516
*Lm*-*18s*	CATG*TCTC*AGTACAAGCCGC	*TC*GGGAC*TC*TGTTTGCATGT	AF370793
*Tc*-*crlf3*	CGATTGTTATGTGGGCGCAGAGAC	GAG*TC*AGTATTGATACGTGTAACA	LOC661093
*Tc*-*α-tubulin*	CGCCAATAACTACGCCAGAG	CGAACGAGTGGAAAA*TC*AAGAA	LOC656649

**Table 2:** PCR program for amplification of *Tribolium castaneum* and *Locusta migratoria crlf3*.

*Step*	*Time (s)*	*Temperature (°C)*	
Initial denaturing	3 min	95	
Denaturing	30	95	30 x
Annealing	30	61	
Elongation	30	72	
Final elongation	5 min	72	

PCR products were run on 1% agarose gels containing Roti®-GelStain (Roth, Karlsruhe, Germany) for 45 min at 75 V. Gels were imaged using the iBright CL1500 Imaging System, (Thermo Fisher Scientific, Osterode am Harz, Germany).

## Results

### Locust Hemolymph Promotes Survival of Hemocytes in Primary Culture

**Figure 1:**
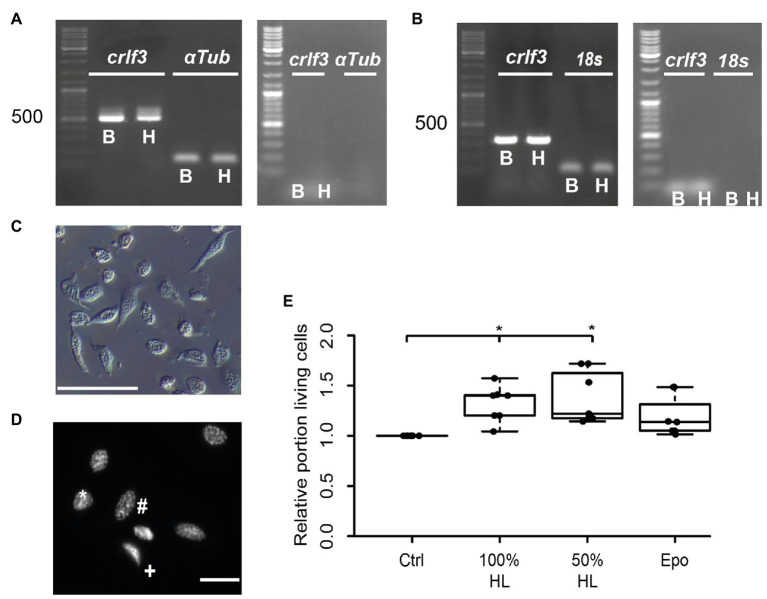
Characterization of hemocyte cultures. **(A)** Reverse transcription polymerase chain reaction (RT-PCR) analysis demonstrates *Tc-crlf3* expression in *T. castaneum* brain tissue (*B*) and hemocytes (*H*). The PCR product has the expected size of 527 bp for *crlf3* amplification. α-*Tubulin* (α*Tub*) was used as control with a predicted amplicon of 128 bp. (−)RT controls were negative (see right gel panel). **(B)** RT-PCR reveals *Lm-crlf3* transcript in *L. migratoria* brain (*B*) and hemocytes (*H*). Both samples show clear bands for *Lm*-*crlf3* at the expected size of 323 bp. *18s rRNA* was amplified as control and generates bands at 135 bp as expected. No gDNA was detected in (−)RT controls (see right panel). **(C)** Locust hemocyte cultures after 7 days *in vitro* contain cells of diverse morphologies. Main hemocyte populations were identified as granulocytes (*) and plasmatocytes (#). Scale bar 50 μm. **(D)** Dapi-labeled nuclei of locust hemocytes. Intact cells display patchy staining reflecting normal chromatin structure (*, #). Dead/dying cells show uniformal nuclear staining (+) indicative of DNA degradation and nuclear condensation. Nuclear morphology allows for characterization of the cell type (^#^phasmatocyte, ^*^granulocyte). Scale bar 10 μm. **(E)** Hemolymph promotes survival of locust hemocytes after 7 days *in vitro*. Both 100% hemolymph and 50% hemolymph diluted in Grace culture medium significantly increase hemocyte survival in comparison to cultures with basal Grace medium. About 33.3 ng/ml erythropoietin (Epo) had no significant effect on hemocyte survival. Statistics were calculated with pairwise permutation test followed by Benjamini Hochberg correction. *n* = 7; Cells evaluated: 154,983. ^*^p < 0.05.

We aimed to study the potential anti-apoptotic effects of locust hemolymph. As a first step, we investigated effects on hemocytes, the cells that naturally are directly exposed to signals that circulate within the hemolymph serum. Hemocyte cultures from *L. migratoria* contained cells with heterogeneous appearance (Figure 1C), many of which could be identified as granulocytes and plasmatocytes. Dapi stainings of hemocytes revealed variable morphologies of nuclei (Figure 1D). Intact and dead/dying hemocytes could be distinguished by chromatin condensation visualized by DNA-associated Dapi fluorescence, as described previously for insect neurons and other cells. CRLF3 has previously been identified as a neuroprotective receptor in *L. migratoria* and *T. castaneum*. RT-PCR analysis revealed expression of *Tc-crlf3* in both brain cells and hemocytes (Figure 1A). Both lanes contained amplicons of expected size (527 bp) which were absent in (−)RT controls. Similarly, *Lm-crlf3* transcripts were detected in both brain tissue and hemocytes of *L. migratoria* (Figure 1B) indicated by amplicons of expected sizes (323 bp). PCR-products related to *crlf3* and *18s rRNA* were absent in (−)RT controls.

We then evaluated the effect of locust hemolymph on hemocyte survival in standard culturing conditions. Locust hemocyte cultures were prepared and maintained for 7 days *in vitro* either in basal Grace medium, pure cell-free locust hemolymph, 50% locust hemolymph diluted in Grace medium or medium supplemented with 33.3 ng/ml Epo. Treatment with either hemolymph concentration significantly increased cell survival in comparison to control cultures (*p* < 0.05, respectively; Figure 1E). Both 100 and 50% hemolymph increased the relative survival to a relative median of 1.4 and 1.2, respectively. Epo did not alter cell survival in comparison to either control or treatment groups.

### Effects of Locust Hemolymph on the Survival of Locust Neurons in Primary Culture

**Figure 2:**
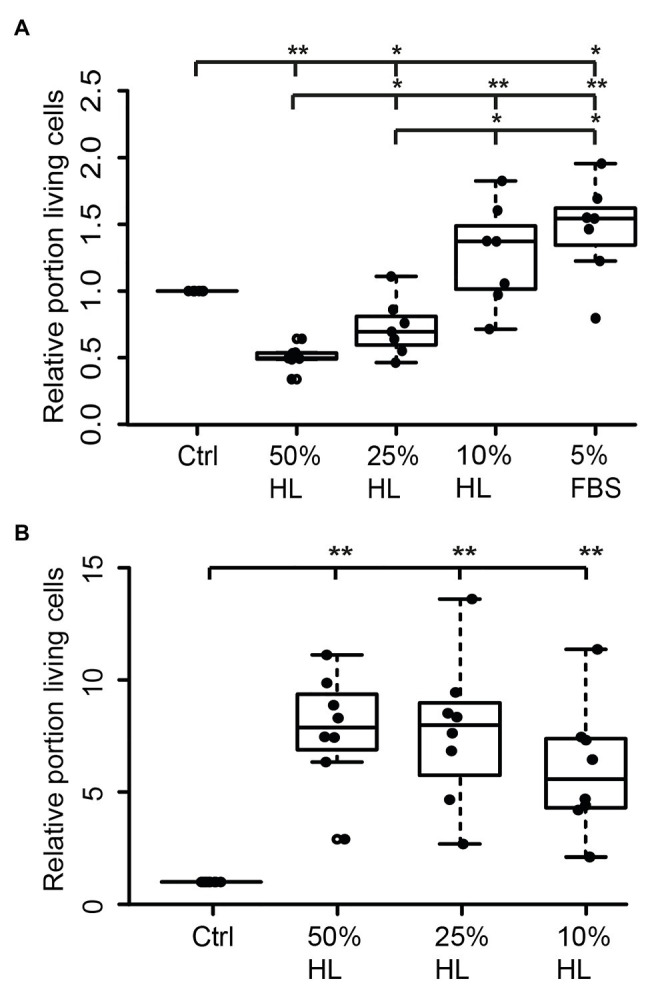
Impact of locust hemolymph on locust neuron survival *in vitro*. **(A)** Relative survival of locust neurons after 2 days *in vitro* with 5% fetal bovine serum (FBS) followed by 3 days in basal L15 culture medium and basal L15 medium supplemented with 5% FBS or 50% hemolymph (HL), 25% HL and 10% HL. About 50 and 25% HL significantly decreased relative survival of neurons in comparison with control. Ten percent HL did not alter neuronal survival. Five percent FBS significantly increased cell survival. *n* = 7; 106,649 cells evaluated. **(B)** Relative survival of locust neurons after 2 days in basal L15 culture medium (without FBS) plus 3 days in basal medium or basalal medium supplemented with 50% HL, 25% HL, and 10% HL. All tested HL supplementations significantly increased relative survival of neurons in comparison to control cultures. *n* = 8; Total cells evaluated: 188,923. Statistics: pairwise permutation test followed by Benjamini Hochberg correction. ^*^p < 0.05; ^**^p < 0.01.

Hemolymph and hemocyte cultures contain a heterologous mix of different cell types that rapidly change their physiological properties in response to pathogens and injury (reviewed in Hillyer and Pass, 2020). Since both hemocytes and brain neurons express CRLF3, we selected the well-characterized neuron culture system for further studies. In a first series of experiments, locust primary brain cell cultures were initially maintained in culture medium supplemented with 5% FBS for 2 days, followed by another 3 days with differing treatments. As expected (and previously reported by Ostrowski et al., 2011), 5% FBS significantly increased neuron survival compared to cultures in basal L15 medium (*p* < 0.05; Median 1.5; Figure 2A). Treatment with HL evoked a dose-dependent effect on relative survival. Fifty percent HL significantly decreased neuron survival in comparison to control cultures (*p* < 0.01) to a median of 0.49. Treatment with 25% HL also decreased cell survival (*p* < 0.05; Median: 0.59), while supplementation of medium with 10% HL did not significantly change cell survival and rather suggested weak promotion of cellular survival (Median 1.02).

Transition of neurons from intact brains into dissociated culture conditions represents a major physiological challenge since it involves disruption of neurites and adjustment to a new environment. FBS supports these adjustments but may initiate prolonged physiological mechanisms that outlast the period of its presence in the culture medium. In a second series of experiments, primary neuron cultures were initially maintained in FBS-free culture medium for 2 days before being exposed to different concentrations of HL for another 3 days. Omitting FBS reduced the total number of surviving neurons in basal L15 medium to less than 5%, compared to ~30% intact neurons in cultures that received FBS treatment during the first 2 days *in vitro*. Exposure of FBS-free cultures to 50, 25, and 10% HL significantly increased relative neuron survival compared to control cultures maintained in basal L15 culture medium (*p* < 0.01 for all HL concentrations tested; Figure 2B). Medians of relative survival were 7.88 for 50% HL, 7.98 for 25% HL, and 5.57 for 10% HL. Although there was a tendency for weaker neuroprotection in culture medium supplemented with 10% HL (compared to 25 and 50% HL), relative survival of neurons was not significantly different between HL concentrations applied.

### Hemolymph Suppresses Hypoxia-Induced Apoptosis in Primary Locust Brain Neurons *via* CRLF3 Activation

**Figure 3:**
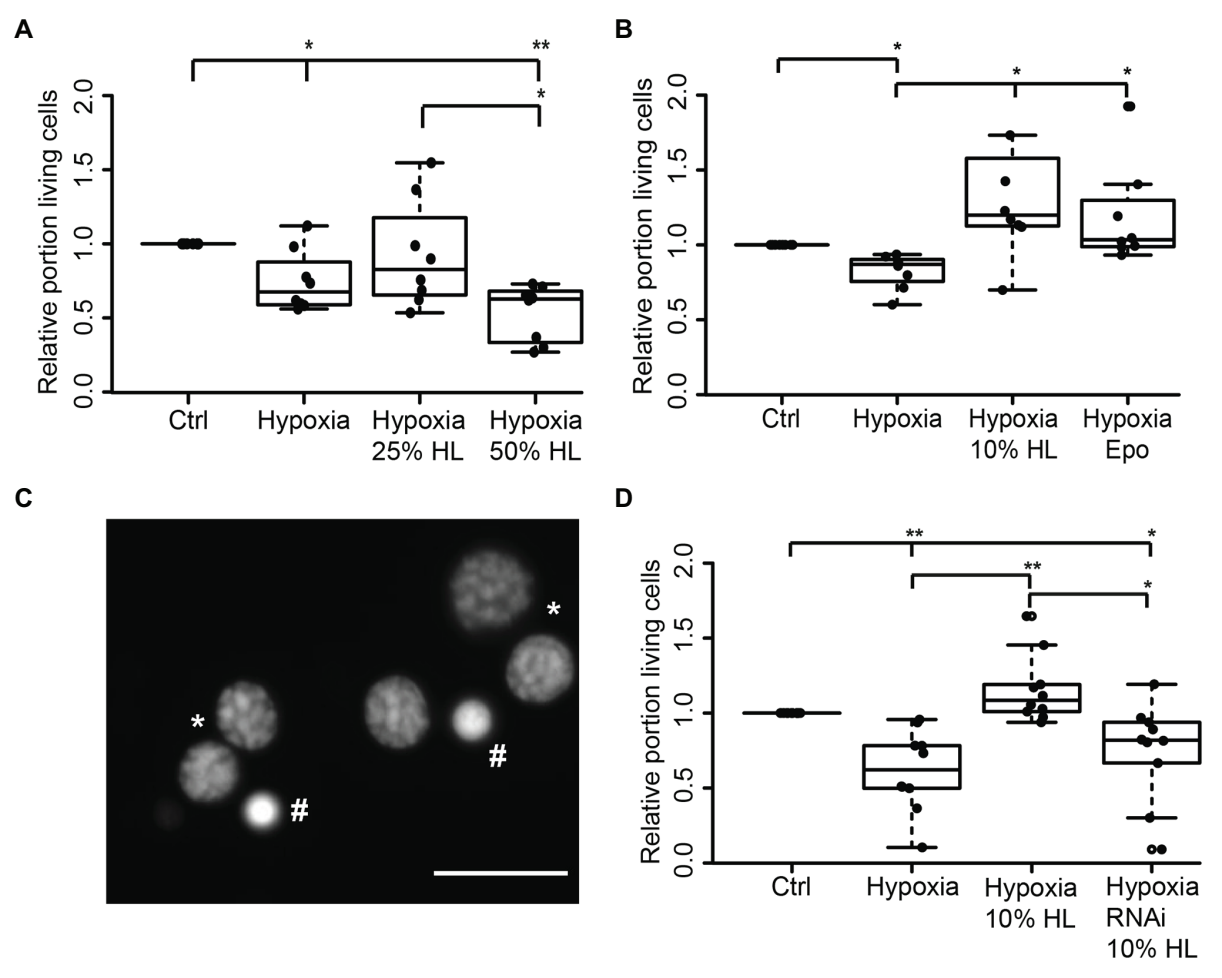
Impact of locust hemolymph on survival of hypoxia-exposed locust neurons *in vitro*. **(A)** Relative survival of hypoxia-exposed (O_2_ < 0.3% for 36 h) primary neurons. Exposure to hypoxia decreased relative survival of neurons in comparison to normoxic control (*p* < 0.05). Twenty-five percent HL did not alter cell survival to either the control nor to the hypoxia group, while treatment with 50% HL significantly decreased cell survival to control cultures and cultures treated with 25% HL (*p* < 0.01 and *p* < 0.05, respectively). *n* = 8; 180,563 cells evaluated. **(B)** Hypoxia-induced cell death (*p* < 0.05 compared with normoxic control) is prevented by 10% HL supplementation (*p* < 0.05 compared with hypoxic control; no difference to normoxic control). Similarly, 33.3 ng/ml Epo increases relative survival in hypoxic conditions to normoxic control levels. *n* = 8; 152,703 cells evaluated. **(C)** Survival of neurons was evaluated on the basis of fluorescent DNA labeling with Dapi. Intact neurons display patchy chromatin labeling (*) while dead/dying cells show uniform staining of condensed chromatin (#). Scale bar 10 μm. **(D)** Locust hemolymph protects neurons from hypoxia-induced apoptosis by activation of cytokine receptor-like factor 3 (CRLF3). Hypoxia significantly (*p* < 0.01) decreased relative survival of neurons compared to normoxic control. Hypoxia-induced cell death was prevented by addition of 10% HL to cell culture medium (*p* < 0.01 compared to hypoxic control). RNAi-mediated knockdown of *crlf3* expression abolished cell protective effects of locust HL on hypoxia-exposed neurons. Relative survival in RNAi- and HL-treated hypoxia-exposed neuron cultures was not different from hypoxic controls and significantly reduced compared with 10% HL-treated hypoxia-exposed cultures (*p* < 0.05). *n* = 10; Cells evaluated: 143,587. Statistics: pairwise permutation test followed by Benjamini Hochberg correction. ^*^p < 0.05; ^**^p < 0.01.

In order to evaluate if HL factors interfere with apoptotic processes, we exposed locust primary neurons to hypoxia (O_2_ < 0.3% for 36 h), which was previously demonstrated to initiate apoptotic cell death (Miljus et al., 2014; Knorr et al., 2020). Based on the results from experiments with FBS-free culture medium (Figure 2B), 50% HL and 25% HL were initially selected for these experiments. As shown in Figure 3A, hypoxia significantly decreased the relative proportion of intact neurons compared with normoxic control cultures (*p* < 0.05). Hypoxia-induced cell death was not prevented by supplementation of culture medium with 25% HL and 50% HL. Fifty percent HL rather further decreased neuronal survival (*p* < 0.01 to control). However, 25% HL was clearly less deleterious for hypoxia-exposed neurons than 50% HL (*p* < 0.05–50% HL) and median relative survival was between normoxic and hypoxic controls (Figure 3A).

Given that the previous experiments suggested a dose-dependent effect of HL treatment toward better survival with lower HL concentrations, the experiments were repeated with 10% HL supplementation. For comparison, additional cultures were treated with 33.3 ng/ml Epo, which was previously shown to prevent hypoxia-induced apoptosis (Miljus et al., 2014; Heinrich et al., 2017; Hahn et al., 2019). Hypoxia significantly decreased cell survival in comparison to normoxic controls in basal L15 culture medium (*p* < 0.05; Figure 3B). Treatment with both Epo and 10% HL rescued cells from hypoxia-induced apoptosis (*p* < 0.05 in both cases) even leading to slightly (though not statistically significant) increased relative neuron survival compared with normoxic control (Medians: Epo 1.03 and 10% HL 1.2). Hence, 10% HL was at least as effective in suppressing hypoxia-induced apoptosis as Epo in its optimal dosage. In order to determine whether HL, like Epo (Hahn et al., 2017, 2019), mediates neuroprotection *via* activation of CRLF3, *Lm-crlf3* expression was knocked down by soaking RNAi. RNAi was achieved by addition of 10 ng/μl dsRNA to the culture medium during the initial 5 days *in vitro*. On day 5, one untreated and the dsRNA-supplemented culture were treated with 10% HL. Together with another untreated neuron culture in basal L15 medium, these cultures were exposed to hypoxic conditions (O_2_ < 0.3% for 36 h) 12 h later. Hypoxia significantly decreased the proportion of intact neurons in comparison to the normoxic control culture (*p* < 0.01; Figure 3D). Treatment with 10% HL prevented hypoxia-induced cell death and significantly increased cell survival (*p* < 0.01 to hypoxic control group) to similar levels as in unchallenged normoxic cultures. RNAi targeting *Lm-crlf3* expression abolished the protective effect of HL. Knockdown of *crlf3* expression reduced relative neuron survival compared to hypoxia-exposed HL-treated cultures (*p* < 0.01; Median 0.82 vs. 1.1) and normoxic control cultures (*p* < 0.05). There was no difference in survival compared to the hypoxic control cultures.

### Effects of Heat-Denatured Hemolymph on Locust Neuronal Survival

**Figure 4:**
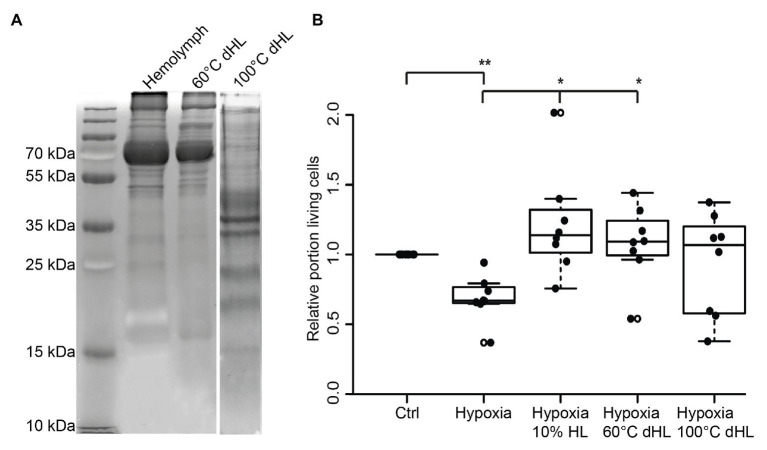
Effects of heat-denatured locust hemolymph (dHL) on relative survival of hypoxia-exposed locust neurons *in vitro*. **(A)** Ten percent sodium dodecyl sulfate–polyacrylamide gel electrophoresis (SDS PAGE) was loaded with 50 μg protein of HL, HL denatured at 60°C and HL denatured at 100°C. HL denatured at 60°C shows similar patterns of separated proteins compared to control HL. In contrast, larger proteins (≥50 kDa) are absent in HL denatured at 100°C. Intense staining of proteins between 30 and 50 kDa (including two clear bands at around 35 kDa) might result from higher concentrations of smaller proteins in the applied sample (50 μg total protein) due to previous removal of heat-denatured proteins. Hundred degrees Celsius dHL proteins show no intense protein bands above 35/40 kDa. **(B)** Locust neurons were treated with either basal L15 medium, 10% HL, 10% 60°C dHL, or 100°C dHL during hypoxia-exposure. Hypoxia significantly decreased relative neuron survival compared to normoxic control. Hypoxia-induced apoptosis was prevented by 10% HL and 10% HL denatured at 60°C. Neuron survival in cultures supplemented with HL denatured at 100°C was not significantly different to normoxic control or hypoxic control cultures. *n* = 8; Total cells evaluated: 160,595. Pairwise permutation test with Benjamini Hochberg correction. ^*^p < 0.05; ^**^p < 0.01.

Assuming that the protective factor contained in locust HL is a peptide or protein, we aimed to gain a rough estimate about its size. In order to do so, we denatured HL at 60 and 100°C before testing its protective effects on hypoxia-exposed neurons. It is expected that larger and more complex proteins are more sensitive to heat-denaturation than smaller proteins (meaning they will be eliminated in HL cooked at 60°C). Following heat-denaturation and removal of precipitated proteins by centrifugation, 50 μg protein from untreated HL, HL denatured at 60°C and HL denatured at 100°C were separated on a 10% SDS-PAGE (Figure 4A). Both untreated and 60°C dHL contained a large portion of proteins in size ranges of ≥55 kDa and showed minor differences of labeling patterns between samples. In contrast, HL denatured at 100°C lacked the larger sized proteins. Increased labeling intensity of smaller-sized proteins (most obvious between ~30 and ~50 kDa) can be noted in HL denatured at 100°C. This effect might be due to the lack of high molecular weight proteins in the sample. In order to determine the neuroprotective functions of heat-denatured HL, locust primary neuron cultures were treated with untreated and heat-denatured HL after 5 days *in vitro*, starting 12 h before hypoxia-exposure for 36 h. Hypoxia significantly decreased cell survival in comparison to untreated normoxic control cultures (*p* < 0.01; Median relative survival 0.67; Figure 4B). Both 10% HL (Median of relative survival 1.13) and 10% HL denatured at 60°C (Median of relative survival 1.07) significantly rescued cells from hypoxia-induced apoptosis (both *p* < 0.05 compared with hypoxia control). Relative neuron survival in cultures supplemented with 10% HL denatured at 100°C (Median 0.81) was neither different from normoxic nor from hypoxic control cultures.

### Locust Hemolymph Protects Beetle Neurons *via* CRLF3 Activation

**Figure 5:**
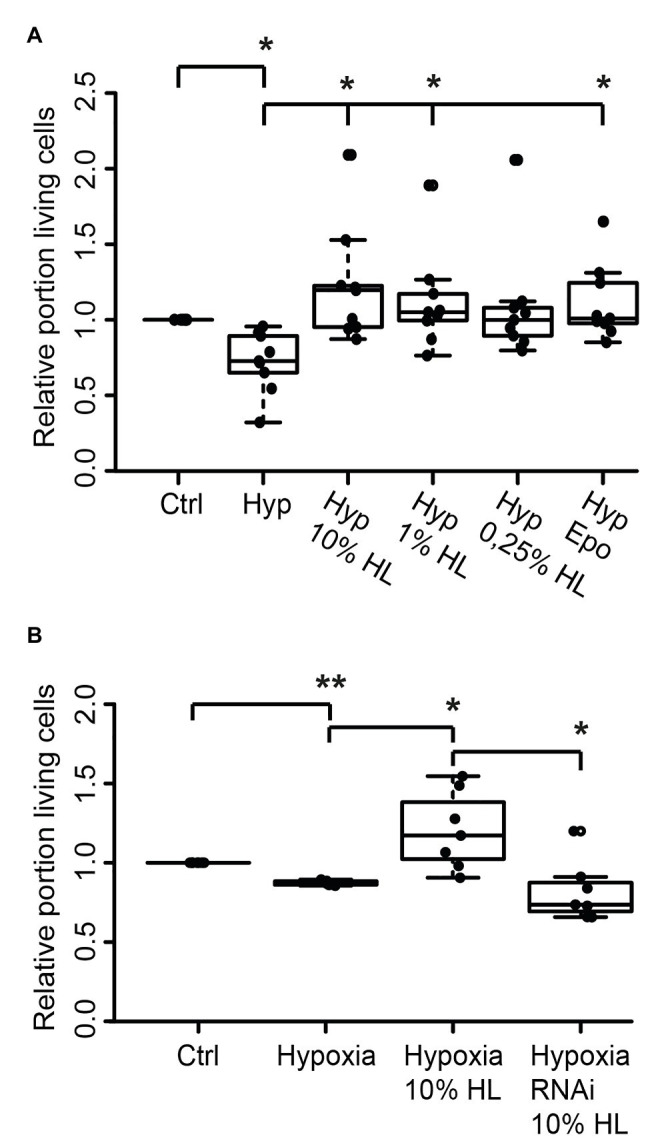
Locust hemolymph protects *Tribolium castaneum* neurons from hypoxia-induced apoptosis *via* activation of CRLF3. **(A)** Hypoxia decreased the relative proportion of intact insect neurons compared to normoxic controls. Ten percent locust HL, 1% locust HL, and 3.33 ng/ml Epo prevented hypoxia-induced death of beetle neurons (*p* < 0.05 compared to hypoxia control, respectively; no difference to normoxic control). About 0.25% locust HL did not significantly alter cell survival compared to normoxic control and to hypoxic control cultures. *n* = 9, total cells evaluated: 185,970. **(B)** *T. castaneum* neurons were maintained for 5 days in standard culture conditions with one culture being exposed to dsRNA targeting *Tc-crlf3*. Hypoxia significantly decreased relative neuron survival in comparison to the normoxic control. The protective effect of 10% locust HL (*p* < 0.05 compared to hypoxic control) was absent after RNAi-mediated depletion of CRLF3 (*p* < 0.05). Relative survival of CRLF3-depleted neurons treated with locust HL was not different from hypoxia-exposed controls without the protective HL supplementation. *n* = 7; Cells evaluated: 163,740. Statistics: pairwise permutation test with Benjamini Hochberg correction. ^*^p < 0.05; ^**^p < 0.01.

Since both *L. migratoria* and *T. castaneum* neurons express CRLF3 that is activated by Epo, we wondered whether the endogenous ligand present in locust hemolymph may also protect beetle neurons. *Tribolium castaneum* primary neuronal cell cultures were established and maintained for 5 days before treatments were initiated. Twelve hours prior to hypoxia exposure (O_2_ < 0.3% for 36 h), neuron cultures were supplemented with either 10, 1, or 0.25% of locust HL or 3.33 ng/ml Epo. As shown in Figure 5A, hypoxia decreased the relative proportion of intact neurons in comparison to normoxic control cultures (*p* < 0.05; Median 0.76). Ten percent locust HL (Median relative survival 1.10) and 1% locust HL (Median relative survival 1.04) significantly increased neuron survival to the level seen in normoxic control cultures (*p* < 0.05 compared with hypoxic control). Treatment with 0.25% locust HL could not significantly protect *T. castaneum* neurons from hypoxia-induced apoptosis. As expected from previous studies (Hahn et al., 2017), Epo protected beetle neurons from hypoxia-induced apoptosis (*p* < 0.05).

We further tested if the neuroprotective effect elicited by locust HL on tribolium neurons was also CRLF3 dependent. *Tc-crlf3* was knocked down by means of soaking RNAi and cells were subsequently treated with 10% locust HL. Hypoxia again significantly reduced cell survival in comparison to untreated controls (*p* < 0.01; Median 0.89; Figure 5B). Treatment with 10% locust HL (Median relative survival 1.28) rescued beetle neurons from hypoxia-induced apoptosis (*p* < 0.05 compared with hypoxic control). The protective effect of locust HL was absent, when *Tc-crlf3* expression in tribolium neurons was suppressed for 5 days prior to the hypoxia challenge (Median relative survival 0.73, *p* < 0.05 compared to HL-treated hypoxia-exposed cultures). Relative neuron survival of CRLF3-depleted cultures was not different from hypoxic control cultures.

## Discussion

The physiological functions of insect hemolymph have been studied in various species and its beneficial effects on cell survival were already noticed in early *in vitro* studies with various cell types (Day and Grace, 1954). Numerous studies have indicated that hemolymph is highly reactive to the physiological state of the insect. The impact of physiological stressors is reflected in the changing molecular and cellular composition of it (Hillyer and Christensen, 2002). Exposure of insects to pathogens or other stressors increased the abundance of a variety of hemolymph proteins, like anti-microbial peptides, prophenol oxidase, apolipophorin, vago, unpaired3, growth blocking peptides, transferrin, and hexamerin (Wang et al., 2007; Welchman et al., 2009; Kingsolver et al., 2013) among others. Principal origin of most humoral defense factors is the fat body. Besides functions in insect metabolism (reviewed in Arrese and Soulages, 2010), it regulates the release and uptake of proteins into and from the serum (Lavine and Strand, 2002; Roma et al., 2010; Hoshizaki, 2013; Duressa et al., 2014). Humoral factors are also produced and secreted from midgut, hemocytes, prothoracic gland, and neurosecretory cells within the *corpora allata*, *corpora cardiaca*, the anterior sympathetic system, ventral ganglia, and various sites along peripheral nerves (Reynolds, 2013; Kodrík et al., 2015). Cytokines and cytokine-like proteins are among these circulating proteins, regulating innate immune responses and adaptive reactions to various stress factors (Beschin et al., 1999, 2001).

Cytokines and their receptors play major roles in immune and stress responses, cell activation, proliferation, maturation, and differentiation in both vertebrates and invertebrates (Shields et al., 1995; Beschin et al., 2001). Apart from some general structural features, cytokines typically share little sequence similarities within and between animal groups (Boulay et al., 2003; Liongue and Ward, 2007). The diversity of cytokines and cytokine receptors identified today most likely date back to ancient molecules in common ancestors of vertebrates and invertebrates (Huising et al., 2006; Liongue and Ward, 2007). CRLF3 is one example for an evolutionarily ancient cytokine receptor, which may be regarded as the prototype for class 1 cytokine receptors (Liongue and Ward, 2007). CRLF3 is highly conserved across eumetazoan species, implying an essential role for the organism (Liongue and Ward, 2007; Hahn et al., 2019). Insect CRLF3, activated by human Epo, initiates anti-apoptotic mechanisms in insect neurons even though insects lack genes for Epo and the classical Epo receptor (Hahn et al., 2017, 2019). Epo mediates anti-apoptotic effects in various mammalian cells (including neurons and erythrocytes) *via* the classical homodimeric Epo receptor and additional tissue-protective alternative Epo receptors (reviewed by Ostrowski and Heinrich, 2018). Several molecules that mimic the cell-protective but not the erythropoietic effects of Epo in vertebrate tissues have been identified (Wrighton et al., 1996; Middleton et al., 1999; Johnson and Jollife, 2000; Brines et al., 2008; Ueba et al., 2010; Wu et al., 2013; Bonnas et al., 2017). Some of these, including the natural Epo splice variant EV-3 and several small peptides with little or no sequence similarity to Epo also protect insect neurons from apoptotic cell death (Miljus et al., 2014; Hahn et al., 2017; own unpublished results). Whether vertebrate CRLF3 may also serve as a tissue-protective receptor for Epo is presently not known.

Given that insects do not express Epo or any known vertebrate Epo receptors, we gained interest in the identification of the endogenous ligand of insect CRLF3. We first confirmed that *crlf3* is expressed in brain and hemocytes of both *L. migratoria* and *T. castaneum*. For both species, PCR amplified fragments of identical size in brain and hemocytes (Figures 1A,B) were confirmed. Next, we exposed primary cultured locust hemocytes for 7 days to rhEpo and cell-free hemolymph collected from different locusts. Supplementation of cultures with pure hemolymph and 50% hemolymph significantly increased cell survival in comparison to serum-free cultures. Epo supplementation only showed a tendency toward support of hemocyte survival which did not reach significance level (*p* = 0.06; Figure 1E). Previous studies on insect (Hahn et al., 2017; Heinrich et al., 2017) and mammalian neurons (Siren et al., 2001; Chong et al., 2003; Weishaupt et al., 2004) demonstrated that Epo elicits neuroprotective effects in an optimum-type curve with both lower and higher concentrations of Epo being less effective. We treated locust hemocytes with 33.3 ng/ml rhEpo, which was determined as the optimal concentration to suppress hypoxia-induced apoptosis in locust primary neuron cultures. It seems that a multiplicity of factors determines the optimal concentration of Epo, within them: species (hypoxia-challenged neurons of *T. castaneum* are best protected by 3.33 ng/ml rhEpo), physiological condition (hemolymph impact on survival of challenged and unchallenged locust neurons discussed below), type of challenge, and cell type. We have not studied the effects of different rhEpo concentrations on primary cultured locust hemocytes. Nonetheless, we predict hemocyte protection to occur with appropriate Epo dosage. However, hemolymph clearly protected primary cultured hemocytes, indicating the presence of protective molecules in hemolymph serum.

Representing the major site of immune defense and the medium that distributes signals and metabolites to all organs, hemolymph is a highly responsive “fluid tissue.” With respect to the surrounding circumstances, both the molecular composition of the serum and the physiological state of various hemocyte types will be altered (Wang et al., 2007; Altincicek et al., 2008; Duressa et al., 2015). In order to reduce this extensive variability, we studied the protective functions of hemolymph serum with well-established protocols of primary neuron cultures from two different species, *L. migratoria* and *T. castaneum*. These were previously utilized to demonstrate the neuroprotective functions of CRLF3 following its activation with rhEpo. However, the hemolymph serum used in our studies was numerously extracted from different locusts (each time pooled from up to 100 individuals received from a commercial breeder) and its molecular composition, including the presence of cell-protective agents, likely varied between these batches. Variability of the cell-protective HL effects described in this study may partly result from pre-exposure of HL-donor animals to stressful conditions (transport, infections, extreme crowding, and others). Keeping locusts under optimal conditions for several days and/or exposing them to a defined stressor before HL extraction may result in a more uniform composition of HL protein content.

We hypothesized that the yet unknown CRLF3 ligand might be an ancestral cytokine with similarity to Epo that acts in a similar, cytoprotective way as Epo does. We demonstrated that locust hemolymph increased the survival of primary-cultured locust neurons in a dose-dependent manner (Figure 2). Compared to serum-free culture medium, addition of locust hemolymph significantly increased neuronal survival with 50 and 25% supplementation showing higher median survival compared to 10% hemolymph. Serum deprivation, containing growth factors and cytokines that support cellular survival in dissociated cultures, has been used as an apoptosis-inducing stressor in various *in vitro* studies with both vertebrate and invertebrate cells (Macleod et al., 2001; Siren et al., 2001; Charles et al., 2005). Though serum-free culturing of adult and embryonic locust neurons has previously been achieved (Kirchhof and Bicker, 1992; Sukiban et al., 2014), only a small portion of brain neurons survive the dissociation process during culture establishment in our study (which involves the disruption of all neurites) without FBS supplementation. The FBS used in our studies has a defined composition of natural components (nevertheless small variations may not fully be excluded) and originated from the same Lot. To increase the number of intact neurons in locust brain cultures, we typically apply FBS during the first day *in vitro*. While promoting neuron survival, FBS is detrimental for locust brain glia (Ostrowski et al., 2011) leading to increasingly pure neuronal cultures during the initial culture period. Since serum has been shown to induce long-lasting effects in cultured neurons, we withdraw FBS from the culture medium 24 h before experimental treatment with hemolymph or Epo.

Serum withdrawal induced cell death in locust and beetle primary neuron cultures while Epo (if applied in appropriate dosage) fully restored the beneficial effects on neuronal survival (Ostrowski et al., 2011; Hahn et al., 2017). Withdrawal of FBS after 2 days significantly decreased neuronal survival during the following 3 days *in vitro* compared to cultures maintained with FBS. This indicates protective effects of FBS throughout prolonged presence in the medium (Figure 2A). In contrast to its effects on neurons that were not supported by additional FBS, hemolymph exposure after 2 days of FBS treatment revealed dose-dependent negative effects (Figure 2B). In this case, higher hemolymph concentrations acted deleterious on neuronal cell survival, while 10% HL showed a trend toward neuroprotection. Moreover, neurons that were initially cultured with FBS and subsequently subjected to hypoxia were best protected by 10% hemolymph. Twenty-five percent hemolymph only weakly (but not significantly) improved survival and 50% hemolymph rather seemed to aggravate hypoxia-induced neuronal death (Figure 3). Together, these results indicate long-lasting effects of FBS that promote neuronal survival beyond its presence in the culture medium. To explain the differing impact of hemolymph on cultured neurons that were exposed to FBS and hypoxia, one has to assume long-lasting changes in the neurons’ physiological state that were induced by different stressful conditions (such as injury during dissociation, presence/absence of serum and other supporting factors, and hypoxia). Depending on their state and the type of challenge, cells may require different amounts and types of protective factors for their survival (Dinarello, 2007). Hence, as observed in our present study, a particular dose of hemolymph (reflecting a certain concentration of ligands) may be beneficial for locust neurons in one situation and deleterious in the other. Studies on *T. castaneum* revealed optimum-type protective effects of different rhEpo concentrations, with high concentrations not only being non-protective but deleterious for neuron survival (Hahn et al., 2017). This indicates that overactivation of CRLF3 and/or its downstream transduction processes has negative impacts on neuron survival. Similarly, protective effects and efficacy of different concentrations of Epo have been shown to vary in murine astrocytes exposed to different inducers of cell death (Diaz et al., 2005) and rat brain neurons after exposure to mild hypoxic periods (Sanchez et al., 2009).

In a previous study with locust primary neuron cultures, *crlf3*-expression was suppressed by soaking RNAi (Hahn et al., 2019). Efficient uptake of dsRNA from extracellular space into insect cells has been associated with two types of cell-surface receptors and clathrin-dependent endocytosis in *Drosophila* (Ulvila et al., 2006). In locusts, supplementation of culture medium with two different dsRNA fragments (non-overlapping targets of *crlf3* mRNA) abolished Epo-mediated protection of hypoxia-exposed neurons completely. This indicated that Epo initiates anti-apoptotic mechanisms by binding to CRLF3 (Hahn et al., 2019). After confirming that 10% hemolymph acted equally protective as 33.3 ng/ml rhEpo on hypoxia-exposed locust neurons (Figure 2B), we selected one dsRNA fragment to knockdown *crlf3*-expression by RNAi. dsRNA-incubation prior to hypoxia exposure abolished the anti-apoptotic effect of 10% hemolymph on locust neurons. This finding suggests that locust hemolymph contains a ligand that activates CRLF3 and its downstream anti-apoptotic pathways (Figure 2D).

Given the high similarity of *crlf3* between *L. migratoria* and *T. castaneum* and the fact that both *Lm*-CRLF3 and *Tc*-CRLF3 mediate neuroprotection upon stimulation with rhEpo (Hahn et al., 2017, 2019), we studied the protective effects of locust hemolymph on beetle neurons. *T. castaneum* neurons were protected from hypoxia-induced apoptosis by 10 and 1% locust hemolymph and the protective effect was clearly mediated by *Tc*-CRLF3 (Figure 5). The protective effect was similar to 3.33 ng/ml rhEpo, which was previously determined as the most protective concentration for *T. castaneum* neurons (Hahn et al., 2017). The most protective Epo concentration for tribolium neurons is only a tenth of the most protective concentration for locust neurons (33.3 ng/ml). In contrast, 10% locust hemolymph protected both locust and beetle neurons to the same degree as the (different) optimal Epo concentrations of both species. One may speculate that the endogenous ligand in locust hemolymph binds to *Lm*-CRLF3 and *Tc*-CRLF3 with similar affinity, whereas rhEpo has a higher affinity to *Tc*-CRLF3. This might result from slight structural differences of the locust receptor to the human and beetle receptor. Amino acid sequences of *Lm*-CRLF3 and *Tc*-CRLF3 share 35% similarity, whereas locust and human CRLF3 display 29% similarity (Hahn et al., 2017, 2019). High conservation and multi-tissue expression of CRLF3 among eumetazoan species suggests an important role for CRLF3-mediated functions in these organisms. The endogenous ligand that circulates within locust hemolymph seems to be conserved among insects and may also be present in species outside the insect clade.

Lepidopteran 30K proteins were shown to retain their protective functions following exposure to 60°C but lost their beneficial effects when exposed to higher temperatures (Kim et al., 2001). A later study described thermostability of 30K proteins up to 70–80°C (Pakkianathan et al., 2015). Heat denaturation disrupts the secondary and tertiary structure of proteins which typically results in the precipitation of denatured proteins from the solvent (Michnik and Drzazga, 2010). Larger and more complex proteins are typically denatured at lower temperatures and are less likely to reassume their native structure during subsequent cooling. Heating locust hemolymph to 60°C had no impact on its anti-apoptotic effects. Heating hemolymph to 100°C resulted in no significant difference in cell viability compared to sole hypoxia exposure, however, the protective activity was retained to some extend (Figure 4). Heating the serum to 100°C removed proteins of ≥50 kDa suggesting that the protective CRLF3 ligand is smaller than this size. Biological activity of a 14 kDa protein Thrombocorticin from sponge was gradually abolished by extending the duration of its exposure to 98°C (Watari et al., 2019), leaving the possibility that longer periods of exposure to 100°C might abolish protective effects of locust hemolymph.

This study indicates the presence of a conserved cytokine in insect hemolymph, activating the phylogenetically conserved CRLF3, whose endogenous ligand has not been identified in any species. Human Epo, its splice variant EV-3 and various small peptides that mimic neuroprotective effects of Epo on mammalian cells all activate insect CRLF3 to initiate anti-apoptotic mechanisms. This suggests some structural similarity between Epo-like ligands and the endogenous CRLF3 ligand present in locust hemolymph. Whether mammalian CRLF3, like insect CRLF3, initiates cell-protective intracellular processes upon Epo binding is currently not known. CRLF3 expression in various insect and mammalian tissues suggests a conserved function in adaptive responses to physiological challenges similar to Epo signaling in mammals. Molecular identification of the insect CRLF3 ligand may lead to the discovery of mammalian orthologues and opportunities to activate beneficial CRLF3 functions for medical treatment.

## Data Availability Statement

The raw data supporting the conclusions of this article will be made available by the authors, without undue reservation.

## Author Contributions

DK and RH designed and supervised the study, and wrote and edited the manuscript. DK, DH, KS, LH, and HP performed the experiments. DK, DH, KS, and LH analyzed the data. All authors contributed to the article and approved the submitted version.

### Conflict of Interest

The authors declare that the research was conducted in the absence of any commercial or financial relationships that could be construed as a potential conflict of interest.

## Abbreviations

Epo: Erythropoietin
rhEpo: Recombinant human Epo
HL: Hemolymph
dHL: Denatured hemolymph
